# Secondhand Smoke Exposure in Primary School Children: A Survey in Dhaka, Bangladesh

**DOI:** 10.1093/ntr/ntx248

**Published:** 2017-12-07

**Authors:** Sarwat Shah, Mona Kanaan, Rumana Huque, Aziz Sheikh, Omara Dogar, Heather Thomson, Steve Parrott, Kamran Siddiqi

**Affiliations:** 1 Department of Health Sciences, University of York, Heslington, York, UK; 2 Department of Economics, University of Dhaka, Social Science Building, Nilkhet, Dhaka, Bangladesh; 3 Centre for Medical Informatics, Usher Institute of Population Health Sciences and Informatics, The University of Edinburgh, Medical School, Teviot Place, Edinburgh, UK; 4 Department of Public Health, Leeds City Council, Leeds, UK

## Abstract

**Introduction:**

We report on second-hand smoke (SHS) exposure based on saliva cotinine levels among children in Bangladesh—a country with laws against smoking in public places.

**Methods:**

A survey of primary school children from two areas of the Dhaka district was conducted in 2015. Participants completed a questionnaire and provided saliva samples for cotinine measurement to assess SHS exposure with a cut-off range of ≥0.1ng/mL.

**Results:**

Four hundred and eighty-one children studying in year-5 were recruited from 12 primary schools. Of these, 479 saliva samples were found sufficient for cotinine testing, of which 95% (453/479) were positive for recent SHS exposure. Geometric mean cotinine was 0.36 (95% CI = 0.32 to 0.40); 43% (208/479) of children lived with at least one smoker in the household. Only 21% (100/479) reported complete smoking restrictions for residents and visitors; 87% (419/479) also reported being recently exposed to SHS in public spaces. Living with a smoker and number of tobacco selling shops in the neighborhood had positive associations with recent SHS exposure.

**Conclusions:**

Despite having a ban on smoking in public places, recent SHS exposure among children in Bangladesh remains very high. There is an urgent need to reduce exposure to SHS in Bangladeshi children.

**Implications:**

Children bear the biggest burden of disease due to SHS exposure than any other age group. However, children living in many high-income countries have had a sharp decline in their exposure to SHS in recent years. What remains unknown is if children living in low-income countries are still exposed to SHS. Our study suggests that despite having a ban on smoking in public places, most primary school children in Dhaka, Bangladesh are still likely to be exposed to SHS.

## Introduction

Worldwide, as many as 40% of children are exposed to second-hand smoking (SHS).^1^ The harms associated with children’s exposure to SHS are now well documented.^2,3^ For the same level of SHS exposure in the environment, children tend to be more susceptible to SHS-related harm due to their higher ventilation rates, than adults.^4^ In terms of disability adjusted life years (DALYs) lost, children bear the biggest burden of disease due to SHS exposure than any other age group.^1^ As children have little control over their environment, they are dependent on others to introduce measures to protect them from SHS exposure.

Recognizing SHS exposure as a public health threat, most countries have introduced comprehensive smoking bans in enclosed public places and workplaces.^5–7^ Since the introduction of these bans, there has been an increase in the number of smoke-free homes in many countries, indicating shifting social norms.^8,9^ Among children, these bans have resulted in a reduction in SHS exposure^10^ and hospital admissions due to respiratory symptoms.^11–14^ However, evidence on the positive impact of smoke-free legislation indicating their successful implementation originated mainly from high-income countries (HIC).^8,15^ In contrast, such evidence remains scarce in low- and middle-income countries (LMICs). The International Tobacco Control (ITC) Evaluation Project, which systematically evaluated implementation of smoke-free policies in public places in several countries, reported poor compliance in the two included LMICs, namely India and Bangladesh.^16,17^ The ITC Evaluation Project assessed the implementation of smoke-free laws in workplaces, public transport, and at homes using self-reported surveys among adults. Children were, however, excluded from these surveys. Moreover, studies reporting an increase in smoke-free homes and a reduction in children’s SHS exposure, since smoking bans in public places in LMICs,^18^ have also relied solely on self-reported measures. Therefore, concerns about the implementation of smoke-free laws remain in many LMICs such as Bangladesh.

Bangladesh was among the first 40 countries that signed the Framework Convention on Tobacco Control (FCTC). The Bangladesh Tobacco Control Act (TCA) 2005, which includes enhanced warning labels on tobacco packaging, smoke-free legislation, and advertising and promotion restrictions was implemented in 2006. It was further strengthened in 2012 with an amendment, including comprehensive smoke-free laws and displaying graphic warning labels. Currently, there is complete prohibition to smoke in the majority of indoor public places, workplaces, and public transport in Bangladesh.^19^ Healthcare and educational facilities are also covered by the legislation with no provision for any outdoor designated smoking zones. In the absence of any baseline figures to compare, it is difficult to say if smoke-free legislation in Bangladesh has had any impact. However, two subsequent waves of postlegislation ITC surveys showed only modest levels of compliance; 49% of participants in 2009 and 37% in 2010 observed smoking in public transport and 46% participants in 2009 and 44% in 2010 reported no smoking restriction rules in their workplace.^20^ On the other hand, prevalence of smoking among men has gone down significantly from 54.0% in 2004 to 44.7% in 2009,^21^ though it is still high compared to the global average of 31.1%.^22^

A recent study based on GATS 2009, Bangladesh reported that an estimated 27.6 million children were exposed to SHS in their homes.^23^ However, this estimate was based on self-reported smoking restrictions at home. We, for the first time, report biochemically verified SHS exposure among school children in Bangladesh. We also explored associations between several sociodemographic and behavioral factors and recent SHS exposure in children.

## Methods

### Design

We conducted a cross-sectional survey of year-5 school children (expected age range 9–11 years) between March and May 2015 in two areas in Dhaka district, Bangladesh. These two areas, Mirpur and Savar, have a population of more than a million each and are geographically representative of urban and semi-urban (agricultural and industrial) contexts, respectively. The survey was part of an on-going pilot randomized controlled trial to assess the effect of a school-based intervention on children’s exposure to SHS.^24^ Therefore, no formal sample size calculation for this survey was done.

### Settings

We first prepared a list of all schools situated within the demarcated areas of Mirpur (49) and Savar (71). We then made phone calls to these schools in phases seeking expression of interest and requesting for an appointment with the head teacher. Those who responded affirmatively within 7 days were subsequently visited. We met the head teachers to brief them about the study. Those consented were assessed for eligibility. Once we reached our required sample size, we stopped calling and assessing more schools. Altogether, we recruited 12 primary schools, six from each area. We included both public and private schools that: followed mainstream curricula approved by the educational authorities; had year-5 classes with >40 and <120 year-5 children per class; and had their own nonsmoking policy. We excluded those who did not have an associated secondary school.

### Participants

We included all children studying in year-5 in the participating schools and were self-reported nonsmokers. Children with mental and physical disabilities; learning difficulties; behavioral problems and/or conduct disorders; and serious medical conditions were excluded. We requested schools to prepare a list of eligible children who were then recruited by obtaining parental consent on an opt-out basis. We also obtained children’s assent through schools.

### Data Collection

The cross-sectional survey consisted of a classroom-administered questionnaire (see web-based Supplementary Questionnaire). A team of researchers distributed paper-based questionnaires to all participating children in their classrooms in-between lessons. The research team attended to any queries and clarifications while children filled in their responses. Following completion of the questionnaires, the research team gave a swab saliva collection kit to each one of the participating children along with a practical demonstration on how to use it. Once collected, all saliva samples were labeled with a unique ID number and transported.

### Measures

#### Tobacco-Related Behavior

Our questions to assess tobacco-related behaviors and attitudes are presented in Box 1. These include self-reported questions about the smoking status of their parents and other adults living in the household, in addition to questions assessing the smoking restriction levels exercised at home, if any. We also assessed their exposure outside homes and in cars. These questions were adapted from those used in previous studies and subsequently tested in a feasibility trial of a smoke-free homes intervention in a Bangladeshi setting.^25^

**Box 1: Some Questions on Smoking-Related Behavior**

1. ‘Does anybody who live with you smoke tobacco?’ (Y/N)
2. ‘Does either of your parents smoke?’ (No/only mother or female carer smokes/only father or male carer smokes/both parents smoke)
3. ‘Does your mother / your father smoke daily?’ (Y/N)
4. ‘Are people who live with you allowed to smoke?’ (Anywhere inside your home/in some rooms in your home/ only in one room in your home/ only outside)
5. ‘Are people who visit your home allowed to smoke’ (Anywhere inside your home/in some rooms in your home/ only in one room in your home/ only outside)
6. ‘Are people who live with you allowed to smoke in front of children?’ (Y/N)
7. ‘Are people who visit your home allowed to smoke in front of children?’ (Y/N)
8. ‘Does anyone smoke while you are in the car?’ (Y/N)
9. ‘Have you been near someone smoking anywhere other than at home or in the car?’ (Y/N)

The variables, smoking restrictions on household members and visitors were later and visitors were later combined to create a composite variable indicating complete restriction if the responses were “yes” to “complete restriction” for both variables versus partial or no restriction.

#### Other Variables

Information was also obtained on age, gender, household asset variables similar to those used in the Demographic & Health Survey (DHS) Bangladesh,^26^ parental education, whether home has any outside space (we defined “open space outside house” as those spaces that are still within the house premises but without a roof, such as the veranda, balcony, yard, garden, lawn, patio, and open roof), number of bedrooms, and number of tobacco selling shops in the neighborhood (within 5 min walking distance). An index of household asset ownership (online Supplementary Table 1) was created as proposed by Morris et al.^27^

#### SHS Exposure

Salivary cotinine, a sensitive biomarker,^28^ which is strongly associated with SHS exposure, was measured. It is the main metabolite of nicotine and has a half-life of approximately 20 h. Levels as low as 0.1 ng/mL can be detected by this method of analysis.^29^ Levels of 0.1ng/mL and above were considered as exposed to SHS.^30^ Saliva samples were tested for cotinine by performing gas-liquid chromatography.^31^

### Statistical Analysis

We conducted a descriptive analysis restricted to those children that provided saliva samples sufficient for cotinine testing. Nondetectable samples were replaced by a value that is half the smallest detected concentration (0.05 ng/mL) before transformation.^32^ Prevalence of SHS exposure across categories of explanatory variables was assessed and compared with those not exposed to SHS. Simple and multiple linear regression analyses were conducted to compute crude and adjusted estimates for the association of variables with cotinine levels. A logarithmic transformation of the salivary cotinine levels was used in the regression analyses due to the skewed nature of this outcome.^33^ Furthermore, sensitivity analyses to assess the influence of observations with cotinine levels >12 ng/mL, indicative of possible tobacco use, on the regression estimates were undertaken by re-running the regressions excluding these observations. In addition, ancillary analyses were carried out on the restricted sample of children living with smoker(s) in the household.

Regression diagnostics were performed to evaluate multicollinearity and influential observations. All analyses were performed using SAS v.9.4 (SAS Institute Inc. Cary, NC) and a level of 0.05 was used for statistical significance.

## Results

We approached 25 schools, seven of which declined to participate due to imminent student examinations and other workload issues. Six schools were ineligible: three did not follow the mainstream curriculum; one had only 15 children in their year-5 class; and two were not associated with secondary schools. There were 576 children studying in year-5 in the 12 participating schools. Out of 484 children present on the day of the survey, 481 consented whereas three did not. These three children did not provide any specific reason for not participating in the study. The age range of 481 children that took part in the study was between 9 and 15 years. All provided saliva samples, of which 99.6% (479/481) samples were found sufficient for cotinine testing. The mean age of participating children was 11.5 years (SD: 0.36) with a sex ratio of 1:1. Table 1 presents SHS exposure status across strata of various explanatory variables.

**Table 1.** Characteristics of 479 School Children According to Recent SHS Exposure

Characteristics	Exposed to SHS *n* = 453 (%)	Not exposed to SHS *n* = 26 (%)	Total *n* = 479 (%)
Sociodemographic factors			
Gender			
Boys	220 (49)	7 (27)	227 (48)
Girls	233 (51)	19 (73)	252 (52)
Maternal education			
No education	69 (15)	2 (8)	71 (15)
Primary	148 (33)	3 (12)	151 (32)
Secondary	167 (37)	9 (35)	176 (37)
Higher education	69 (15)	12 (46)	81 (17)
Paternal education			
No education	42 (9)	1 (4)	43 (8)
Primary	115 (26)	2 (8)	117 (25)
Secondary	165 (37)	7 (26)	172 (36)
Higher education	131 (29)	16 (62)	147 (31)
Home has outside space			
Yes	303 (67)	22 (85)	325 (68)
No	150 (33)	4 (15)	154 (32)
Smoking-related behaviors			
Lives with smoker/s			
Yes	198 (44)	10 (38)	208 (43)
No	255 (56)	16 (62)	271 (57)
Smoking restrictions to both household smokers and visitors			
Complete restriction	94 (21)	6(23)	100 (21)
Partial or no restriction	359 (79)	20 (77)	379 (79)
Visitors allowed to smoke in front of children			
Yes	77 (17)	3 (12)	80 (17)
No	376 (83)	23 (88)	399 (83)
Someone smokes inside car			
Yes	212 (47)	12 (46)	224 (47)
No	241 (53)	14 (54)	255 (53)
Near someone smoking other than home and car			
Yes	394 (87)	25 (96)	419 (87)
No	59 (13)	1 (4)	60 (13)
Analysis restricted to children living with smoker/s (*n* = 208)			
	198 (95)	10 (5)	208
Parental smoking			
Neither parent smokes	2 (1)	0	2 (1)
Only mother/female carer smokes	1 (0.5)	0	1(0.5)
Only father/male carer smokes	194 (98)	10 (100)	204 (98)
Both parents smoke	1 (0.5)	0	1 (0.5)
Parent/s smoke everyday			
Yes	183 (92)	10 (100)	193 (93)
No	15 (8)	0	15 (7)
Smoker allowed to smoke in front of children			
Yes	93 (47)	6 (60)	97 (47)
No	105 (53)	4 (40)	111 (53)

Based on salivary cotinine, overall 95% (453/479; 95% CI = 92.2 to 96.4) children were found to have recent exposure to SHS including 0.6% (3/479; 95% CI = 0.13 to 1.8) children who were considered possible tobacco users due to their cotinine levels higher than 12ng/mL. Only 21% (100/479; 95% CI = 17.3 to 24.5) children reported complete smoking restriction for both residents and visitors but 94% (94/100; 95% CI = 87.4 to 97.8) of these children were still found to be recently exposed to SHS. Of all participants, 43% (208/479; 95% CI = 38.9 to 48) reported living with smoker(s), of whom 98% (204/208; 95% CI = 95.2 to 99.5) reported that only their father/male carer smoked. Out of these, 94% (192/204; 95% CI = 89.9 to 96.9) reported that father/male carer smoked every day. Out of 208 children who lived with smoker/s, 95% (198/208; 95% CI = 91.3 to 97.7) were found to be recently exposed to SHS.

Table 2 presents geometric mean cotinine, bivariate and multiple linear adjusted estimates of SHS exposure across categories of explanatory variables. The arithmetic and geometric mean of the overall sample were (0.98 ng/mL; SD: 6) and (0.36; 95% CI = 0.32 to 0.40 ng/mL), respectively. Based on the unadjusted regression models, girls compared to boys (ß = 0.75; 95% CI = 0.62 to 0.90); children whose parents had secondary (ß = 0.61; 95% CI = 0.46 to 0.79 for maternal education) and higher education (ß = 0.44; 95% CI = 0.32 to 0.61 for maternal and ß = 0.49; 95% CI = 0.35 to 0.69 for paternal education) compared with no education; and households with high SES (ß = 0.98; 95% CI = 0.97 to 0.99) had statistically significant lower cotinine levels. There was a 12% decrease in geometric mean cotinine for each additional bedroom; and 5% increase in geometric mean cotinine for each additional tobacco selling shop in the neighborhood. Children living with smoker(s) had geometric mean cotinine value (ß = 1.90; 95% CI = 1.60 to 2.29) approximately double the mean value among those not living with smoker(s). This explained about 12% of the variability in SHS exposure. Multiple linear regression yielded statistically significant negative association of logarithmic cotinine with SES, maternal education, and positive association with living with smoker(s) and number of tobacco selling shops in the neighborhood after adjusting for all other factors.

**Table 2.** Regression Analysis of Explanatory Variables of Cotinine Levels (Log) in Nonsmoking Primary School Children

Variable		Mean cotinine^a^	Regression coefficients (unadjusted)				Regression coefficients (adjusted*)				
			Exp estimate^b^	SE	95% CI		Exp estimate^b^	SE	95% CI		*p* value
Saliva cotinine levels		0.36	**—**		**—**						
Socioeconomic and geographic factors											
Gender	Boys	0.42	—		—	—	—		—	—	
	Girls	0.32	0.75	0.15	0.62	0.90	0.86	0.08	0.73	1.02	.10
Maternal/female carer education level	No education	0.53	—		—	—	—		—	—	
	Primary	0.43	0.81	0.14	0.61	1.07	1.11	0.14	0.68	1.20	.47
	Secondary	0.32	0.61	0.14	0.46	0.79	0.73	0.15	0.54	0.98	.04
	Higher education	0.24	0.44	0.16	0.32	0.61	0.69	0.19	0.62	0.48	.001
Paternal/male carer education level	No education	0.50	—		—	—	—		—	—	
	Primary	0.49	0.82	0.18	0.69	1.39	1.16	0.18	0.83	1.65	.38
	Secondary	0.37	0.74	0.17	0.53	1.03	1.07	0.18	0.64	1.52	.70
	Higher education	0.25	0.49	0.17	0.35	0.69	0.88	0.19	0.59	1.28	.50
SES		—	0.98	0.01	0.97	0.99	0.98	0.01	0.97	0.99	0.02
Environmental											
Home has any outside space	No	0.45	—		—	—	—		—	—	
	Yes	0.32	0.72	0.10	0.59	1.14	0.84	0.09	0.69	1.01	.28
Number of bedrooms			0.88	0.04	0.80	0.95	0.96	0.04	0.88	1.04	0.29
Number of tobacco selling shops in the neighborhood			1.05	0.01	1.03	1.08	1.04	0.01	1.02	1.07	0.001
Smoking-related behaviors											
Lives with smoker	No	0.27	—		—	—	—		—	—	
	Yes	0.52	1.90	0.08	1.60	2.29	2.08	0.12	1.62	2.66	<.0001
Smoking restrictions to both residents and visitors	Complete restriction	0.48	—		—	—	—	—	—	—	
	Partial and/or no restriction	0.34	0.70	0.12	0.56	0.89	1.26	0.15	0.94	1.68	.13
Anyone smokes inside car	No	0.37	—		—	—					
	Yes	0.36	0.98	0.09	0.81	1.17	1.03	0.09	0.87	1.23	.71
Near someone smoking other than home and car	No	0.44	—		—	—	—	—	—	—	
	Yes	0.35	0.80	0.14	0.61	1.06	0.83	0.13	0.63	1.07	.16

^a^Observed geometric mean cotinine.

^b^Regression coefficients have been exponentiated to represent multiplicative effect on cotinine levels associated with unit increase in possible predictor variables. For categorical predictors, it describes a multiplicative change compared with the reference category.

*Estimates of SHS exposure for each variable while adjusting for all other variables in the model.

Approximately 27% of the overall variation in logarithmic cotinine was explained by the multiple regression model, which included all the explanatory variables included in the unadjusted regression models.

In the analysis restricted to children living with smoker(s) in the household, multiple regression analysis controlling for other explanatory variables showed an independent negative association between SES and logarithmic cotinine (data not shown here but provided in the online Supplementary Table 2). There was weak evidence that complete smoking restriction to smokers living in the household and to visitors and whether smoking was allowed in front of children were associated with logarithmic cotinine.

Multiple regression sensitivity analysis excluding the three possible tobacco users showed similar results as the overall group, see Supplementary Table 3 for further details. Although geometric mean cotinine levels demonstrated a dose response relationship across maternal and paternal education levels, it was borderline significant when adjusted for other explanatory factors in the model.

## Discussion

To our knowledge, this is the first study reporting biochemically validated SHS exposure among children in a LMIC. We also explored several socioeconomic and behavioral variables and their association with recent SHS exposure. Our findings suggest that the prevalence of SHS exposure in children living in Dhaka district, Bangladesh could be as high as 95%. Overall, our sample had high average cotinine levels (0.36 ng/mL); 0.50 ng/mL in those living with smokers compared with 0.26 ng/mL in those not living with smokers. Our findings are most likely to be explained by a combination of high proportions of smokers living in children’s homes and/or communities with little or no smoking restrictions. With 80% children reporting social visibility of smoking in their surrounding public spaces, it is likely that these children got exposed to SHS in public places as well as or instead of their homes and cars. We did not ask about which public places did they feel they got exposed to tobacco smoke but based on our knowledge of children’s activities outside homes in Bangladesh, these are likely to be public transport and shops. Given that smoke-free legislation has also been in place for over a decade in Bangladesh, we did not anticipate finding 95% children with recent SHS exposure in our sample. This to a great extent might reflect poor compliance to and enforcement of smoke-free laws in public places and unhealthy smoking behaviors inside homes, contributing to a large proportion of children being exposed to SHS. Moreover, among those who were recently exposed to SHS, almost a quarter reported complete smoking restrictions at home. This is in line with the World Health Organization (WHO) report stating that separate smoking rooms and ventilation systems are not effective in preventing SHS exposure.^34^ About 43% of children reported living with a smoking parent: the father/male carer in 99% of cases. Less than a quarter of all children and only half of those living with smoker(s) reported complete smoking restrictions at home.

Our estimates on recent SHS exposure in primary school children in Dhaka are more than double of those expected globally (40%)^1^ and in stark contrast with those reported in the UK (31%) and in Canada (9.2%),^35,36^ high-income countries (HICs) with comprehensive smoke-free legislation. Mean cotinine levels were also higher (0.36 ng/mL) than those observed in a nationally representative sample in England (0.11 ng/mL) in 2012.^35^ However, in contrast to the data from England obtained from the National Health Survey including children aged 9–15 years, our sample was not nationally representative. England has seen a steady and substantial decline in the proportion of children whose parents were reported being current smokers (41% in 1998 to 28% in 2012); 87% children in England now live in smoke-free homes. Even among those who lived with parents who smoke in 2012, 61% lived in a smoke-free home and 30% had undetectable cotinine. The Canadian Community Survey also reported marked decline (12.6% in 2011 to 9.2% in 2014) in the proportion of children exposed to SHS at home. A lot of this decline in SHS exposure is attributed to the successful implementation of smoke-free laws that received an overwhelming support among general public (both smokers and nonsmokers).^37^ In Bangladesh, smoke-free policies have existed for more than a decade now; but in contrast to many HICs, public support for such legislation has been relatively lukewarm (44% in both smokers and nonsmokers).^20^

Among other findings, nearly half of the children in our sample were living with at least one smoking resident. This is similar to the findings of a previous survey conducted in the same localities of Dhaka district.^38^ The above survey also reported that 64% of households with at least one smoker reported no smoking restrictions.^38^ Based on GATS 2009 Bangladesh data, 57% households had only partial or no smoking restrictions.^39^ On the other hand, 79% of children in our study reported living in households with only partial or no smoking restrictions. A possible explanation for this difference could be that the two previous studies were based on adult self-reports whereas ours was based on children’s self-reports. In our study, most children also reported SHS exposure in public places. This is in line with the Global Youth Tobacco Survey (GYTS) Bangladesh, which showed a self-reported exposure of youth to SHS in 42% in 2007^40^ and 59% in 2013.^41^ Compared to this, in neighboring India, only 37% of youth reported being around others who smoke in places outside their homes.^42^

Our findings are generally in keeping with published literature, which shows parental smoking behavior and family’s socioeconomic position as the two key correlates of the domestic exposure of children to SHS.^43–45^ We were unable to detect any association between children’s salivary cotinine levels and self-reported smoking restrictions in their homes. This finding is at odds with some other studies from HICs.^46^ On the other hand, living with smoker(s), which in almost all cases was the father/male carer, was associated with children’s salivary cotinine levels. It is possible that the smoking status of the adults in the home determined both the level of smoking restrictions and the resulting level of recent SHS exposure in children. There is some support to this idea by the marginally significant association found between smoking restrictions and the salivary cotinine levels of those children that were living with smokers. Another explanation is that this could be due to a vast number of children in our sample reported being recently exposed to tobacco smoke outside their homes. Among others, maternal education, SES, and the number of tobacco selling shops in the neighborhood were found to have significant association with recent SHS exposure. Large differences in general tobacco use between different social strata could partly explain similar differences between children’s SHS exposure at home. For an equal smoking prevalence, further differences in implementing household smoking restrictions have also been reported between different social classes.^47^

Our study had some limitations. Our cross-sectional survey was based on a nonrandom relatively small sample of children drawn from primary schools in two areas of Dhaka. This is not representative of the national picture, since it does not cover other districts of Bangladesh and also does not include children not attending schools. However, the two study areas did represent the typical urban and semi-urban settings in and around Dhaka. There is a possibility that children participating in our study underreported smoking behaviors such as complete smoking restriction at home. Children could have been exposed to SHS while being completely unaware of smoking inside their homes in a specific room/area away from them. Although we included questions like parental education and the number of cigarette outlets in the neighborhood, we acknowledge that the responses to these items in a self-reported children survey would have questionable validity. Being focused on SHS exposure at homes, we did not ask more detailed questions on exposure outside homes. Further self-reported questions might have pointed out to specific sources of SHS exposure among public spaces.

Objective measurement of SHS exposure at the population level is an important surveillance tool for tobacco control and should be incorporated within national health surveys (tobacco specific or otherwise). For Bangladesh, this can provide reliable prevalence estimates and future trends of SHS exposure in children. If validation of self-reported SHS exposure among children could be achieved, then future SHS exposure surveillance activities could rely on self-reports only. Our study highlights that current tobacco control measures in Bangladesh are unable to protect a vast majority of children from SHS exposure. We are concerned that this exposure is contributing towards children’s poor health and development in Bangladesh. We recommend a four-pronged approach to deal with this issue, namely to: (1) run public media campaigns to raise awareness about the harms of SHS exposure to children, in particular and gauge public support for smoke-free public spaces; (2) enforce implementation and monitoring of smoke-free laws in public spaces using statutory authorities; (3) work with service industry, transport, and other major corporations, both in public and private sector, in order to implement smoke-free laws in their jurisdiction; and (4) to develop, evaluate and implement nonlegislative interventions such as smoke-free homes to encourage families and communities to implement voluntary restrictions on smoking in private homes and cars. Moreover, children whose parents smoke, especially those from socially disadvantaged families, should be recognized as key target groups for such tobacco control measures.

## Funding

This study was conducted as part of CLASS II trial funded by Medical Research Council, UK (MR/M020533/1).

## Declaration of Interests

*None declared*.

## Supplementary Material

Supplementary Questionnaire

Supplementary Tables
