# Supplementary material for: Secondhand Smoke Exposure in Primary School Children: A Survey in Dhaka, Bangladesh
Source: Nicotine Tob Res. 2017 Dec 7;21(4):416–23. doi: 10.1093/ntr/ntx248 (PMC6472694; doi:10.1093/ntr/ntx248)
Supplement: Supplementary Questionnaire [file ntx248_suppl_supplementary_questionnaire.docx]

# Children Learning About Second-hand Smoke

# CLASS II trial

**Survey**

To be completed by the child who is eligible and has given consent to participate in the trial

*For office use only:*

1. **Division ID:……/……..**
2. **Cluster ID:……/……..**
3. **Participant ID:……./……./…….**

| **INSTRUCTIONS FOR COMPLETING THIS SURVEY** |
| --- |

Thank you very much for agreeing to take part in the CLASS II trial

- Please do not put your name on this paper.
- Your answers are confidential.
- Answer every question you can.
- If you are unable to understand a question, ask your teacher or research team for clarification
- If you are unable to answer a question or if you would rather not answer a question, leave it out and go on to the next one.
- For most questions, there is a choice of answers. Pick the one that is true for you and place a cross in the box next to it.

For example: Yes

No

- For some questions, you may need to write a short answer or number in the space provided
- These questions are about tobacco smoke; this includes smoke from cigarettes, bidi, pipes, sheesha, waterpipes, hookah, nargileh and cigars.

**There are 3 Sections in this Survey:**

**Section A: Questions about yourself and your household**

**Section B: Questions about smoking**

**Section C: Questions about your health**

**PLEASE COMPLETE THIS SURVEY USING A BLACK OR BLUE BALLPOINT PEN**

Please enter the date you competed this Survey:…………../………../…………….

*Day Month Year*

**SECTION A: QUESTIONS ABOUT YOURSELF AND HOUSEHOLD**

1. Your date of birth

…………/………./…………

Day Month Year

1. Please indicate your gender

[ ] Male

[ ] Female

1. Does your home have any outside space (e.g. garden, yard, balcony, veranda etc.)?

[ ] Yes

[ ] No

1. How many bedrooms are there in your home (excluding bathroom, kitchen, conservatory etc)?

*Please write a number*

*………./………*

1. How many adults (over 18) live with you?

*Please write a number*

*………./………*

1. How many children (18 and under) live with you?

*Please write a number*

*………./………*

1. Please tell me whether your household have the following items ( DHS Bangladesh)
2. Electricity

[ ] Yes

[ ] No

1. Flush toilet

[ ] Yes

[ ] No

1. Fixed telephone

[ ] Yes

[ ] No

1. Cell telephone

[ ] Yes

[ ] No

1. Television

[ ] Yes

[ ] No

1. Radio

[ ] Yes

[ ] No

1. Refrigerator

[ ] Yes

[ ] No

1. Car

[ ] Yes

[ ] No

1. Moped/Scooter/Motorcycle

[ ] Yes

[ ] No

1. What type of fuel is used for cooking? (DHS Bangladesh)

[ ] Electricity

[ ] LPG/natural gas/biogas

[ ] Kerosene

[ ] Coal/charcoal

[ ] Wood

[ ] Straw/shrubs/grass

[ ] Agricultural crops

[ ] Animal dung

[ ] Other

1. In your home, are there
2. Water damage

[ ] Yes

[ ] No

1. Damp stains or paint peeling

[ ] Yes

[ ] No

1. Visible mold

[ ] Yes

[ ] No

1. Mold odor

[ ] Yes

[ ] No

1. In your home are there cattle (bulls, buffaloes or cows)/pets/poultry (chicken or ducks) etc.?

[ ] Yes

[ ] No

1. What level of education did your mother/female carer complete?

[ ] No education

[ ] Primary

[ ] Secondary

[ ] Higher education

1. What level of education did your father/male carer complete?

[ ] No education

[ ] Primary

[ ] Secondary

[ ] Higher education

1. Approximately how many shops that sell cigarettes or tobacco are in your neighborhood (within 5 minutes walking distance of your home)?

*Please write number*

………/……..

**SECTION B: QUESTIONS ABOUT SMOKING**

1. Does anybody who lives with you smoke tobacco?

[ ] Yes

[ ] No ***Go to Question 21***

1. Think about all the people that live with you now, including yourself
2. How many adults (over 18) smoke?

*Please write number*

………/……...

1. How many children (18 and under) smoke?

*Please write number*

………/………

1. Does either of your parents smoke?

[ ] No ***Go to question 19***

[ ] Only mother/female carer smokes ***Go to question 17***

[ ] Only father/male carer smokes ***Go to question 18***

[ ] Both parents smoke ***Go to question 17***

1. Does your mother or female carer (e.g. grandmother/step mother) smoke daily?

[ ] Yes

[ ] No

[ ] Don’t know

1. Does your father or male carer (e.g. grandfather/step father) smoke daily?

[ ] Yes

[ ] No

[ ] Don’t know

1. Are people who live with you allowed to smoke?

[ ] Anywhere inside your home

[ ] In some rooms in your home

[ ] Only in one room in your home

[ ] Only outside

1. Are people who live with you allowed to smoke in front of children?

[ ] Yes

[ ] No

1. Are people who visit your home allowed to smoke?

[ ] Anywhere inside your home

[ ] In some rooms in your home

[ ] Only in one room in your home

[ ] Only outside

1. Are people who visit your home allowed to smoke in front of children?

[ ] Yes

[ ] No

1. Think about when you travel in a car. Does anyone smoke while you are in the car?

[ ] Yes

[ ] No

1. Have you been near someone smoking anywhere other than at home or in the car?

[ ] Yes

[ ] No

If you crossed yes, please write where else you have been near someone smoking

………………………………………………………………………………………

1. Have you ever smoked tobacco?

[ ] Yes

[ ] No ***Go to question 28***

1. Think about the **last 30 days**. On how many of these days did you smoke?

*Please put a number*

………./………

1. Over **your whole life** about how many cigarettes/bidis/shisha/huqqa have you smoked?

*Please put a number*

………/……..

1. Have you ever tried or experimented with tobacco smoking, even a few puffs?

[ ] Yes

[ ] No

1. Do you think that you will try tobacco smoking soon?

[ ] Yes

[ ] No

1. If one of your best friends were to offer you to smoke tobacco, would you smoke it?

[ ] Definitely yes

[ ] Probably yes

[ ] Probably not

[ ] Definitely not

1. Do you think you will be smoking tobacco **1 year from now**?

[ ] Definitely yes

[ ] Probably yes

[ ] Probably not

[ ] Definitely not

**PART C: QUESTIONS ABOUT YOUR HEALTH**

1. Have you experienced any of the following recently (in the last week)?
2. Cough on waking

[ ] Yes

[ ] No

1. Wheeze on waking

[ ] Yes

[ ] No

1. Cough during the day

[ ] Yes

[ ] No

1. Wheeze during the day

[ ] Yes

[ ] No

1. Shortness of breath during the day

[ ] Yes

[ ] No

1. Night cough

[ ] Yes

[ ] No

1. Wheeze or shortness of breath during the night

[ ] Yes

[ ] No

1. Runny nose or sneezing

[ ] Yes

[ ] No

1. Blocked or stuffy nose

[ ] Yes

[ ] No

1. Sore throat or hoarse voice

[ ] Yes

[ ] No

1. Headaches or face aches

[ ] Yes

[ ] No

1. Aches or pains elsewhere

[ ] Yes

[ ] No

1. Feeling chill/fever or shivers

[ ] Yes

[ ] No

1. Are you currently taking any medications?

[ ] Yes

[ ] No

1. If you said “yes”, is it for?

[ ] Asthma

[ ] Other condition

**Describing your health TODAY**

1. Under each heading, please tick the ONE box that best describes your health TODAY

**Mobility** (walking about)

[ ] I have no problems walking about

[ ] I have some problems walking about

[ ] I have a lot of problems walking about

**Looking after myself**

[ ] I have no problems washing or dressing myself

[ ] I have some problems washing or dressing myself

[ ] I have a lot of problems washing or dressing myself

**Doing usual activities** (*e.g. going to school, hobbies, sports, playing, doing things with family or friends*)

[ ] I have no problems doing my usual activities

[ ] I have some problems doing my usual activities

[ ] I have a lot of problems doing my usual activities

**Having pain or discomfort**

[ ] I have no pain or discomfort

[ ] I have some pain or discomfort

[ ] I have a lot of pain or discomfort

**Feeling worried, sad or unhappy**

[ ] I am not worried, sad or unhappy

[ ] I am a bit worried, sad or unhappy

[ ] I am very worried, sad or unhappy

These questions ask about contact with different health services. Please read the information below before you answer the questions:

**Outpatient** visits are for medical procedures or tests that can be done in a medical centre without an overnight stay. The person would not be having any form of surgery/operation. Examples of outpatient visits include: podiatry clinics, mental health clinics, orthodontists, ophthalmologists, blood tests, children's health centre, diagnostic tests. They do not include visits to your GP.

**Inpatient** visits require an overnight stay in hospital

**Day case hospital** visits are for minor operations which would normally take most of the day but do not require an overnight stay in hospital.

Number of **Prescriptions** Please include the total number of items on all prescriptions obtained over the period stated.

Please write the number of visits/contacts you have had for a personal health problem with each service (given below) during the **last three months.**

Number of appointments with Doctor practicing at health center/clinic/hospital

………../……….

Number of appointments with doctor at home

………../……….

Number of appointments with Nurse at health center/clinic/hospital

………./……….

Number of appointments with Nurse at home

………../………..

Number of outpatient visits

………../………..

Number of admissions/hospitalisation

………../………..

Number of emergency visits

………../………..

Number of prescriptions

………../………..

**Thank you for completing this survey. Please return it to your class teacher**
