# Supplementary material for: Secondhand Smoke Exposure in Primary School Children: A Survey in Dhaka, Bangladesh
Source: Nicotine Tob Res. 2017 Dec 7;21(4):416–23. doi: 10.1093/ntr/ntx248 (PMC6472694; doi:10.1093/ntr/ntx248)
Supplement: Supplementary Tables [file ntx248_suppl_supplementary_tables.docx]

| **Table 1 SES index created from the household asset variables as proposed by Morris et al** | | | | | | | | | | |
| --- | --- | --- | --- | --- | --- | --- | --- | --- | --- | --- |
| **Obs** | **Electricity** | **Flush toilet** | **Fixed Telephone** | **Cell phone** | **Television** | **Radio** | **Refrigerator** | **Car** | **Moped/scooter** | **SES index** |
| 1 | Yes | No | No | Yes | Yes | No | Yes | No | No | 4.5457 |
| 2 | Yes | No | No | Yes | No | No | Yes | No | No | 3.4219 |
| 3 | Yes | No | No | Yes | Yes | No | Yes | No | No | 4.5457 |
| 4 | Yes | No | No | Yes | Yes | No | Yes | No | No | 4.5457 |
| 5 | Yes | No | No | Yes | Yes | No | Yes | No | No | 4.5457 |
| 6 | Yes | No | No | No | No | Yes | No | No | No | 6.4785 |
| 7 | Yes | No | No | Yes | Yes | No | Yes | No | No | 4.5457 |
| 8 | Yes | No | No | Yes | Yes | No | Yes | No | Yes | 15.2346 |
| 9 | Yes | No | No | Yes | Yes | No | Yes | No | No | 4.5457 |
| 10 | Yes | No | No | Yes | Yes | Yes | Yes | No | No | 10.0116 |
| 11 | Yes | No | No | Yes | Yes | No | Yes | No | No | 4.5457 |
| 12 | Yes | Yes | No | Yes | Yes | No | Yes | No | No | 8.3331 |
| 13 | Yes | Yes | No | Yes | Yes | No | Yes | No | No | 8.3331 |
| First 13 observations from the data set to present the values of SES index created from the household asset variables as proposed  by Morris et al which is to assign each item in the list of assets a weight equal to the reciprocal of the proportion  of the households who own one or more of that item, then multiplying that weight by the number of units of asset  owned by the household, and summing the product over all possible assets | | | | | | | | | | |

| **Table 2 Regression analysis of explanatory variables of SHS exposure (log cotinine) in non-smoking primary school children: restricted to children living with smoker/s in household** | | | | | | | | | | | |
| --- | --- | --- | --- | --- | --- | --- | --- | --- | --- | --- | --- |
| **Variable** | | **Mean cotinine^a^** | **Regression coefficients (unadjusted)** | | | | **Regression coefficients (adjusted)*** | | | | **P value** |
|  |  |  | **Exp estimate^b^** | **SE** | **95% CI** | | **Exp estimate^b^** | **SE** | **95% CI** | |  |
| Saliva cotinine levels | | 0.52 | **-** | **-** | **-** | | **-** | **-** | **-** | **-** | **-** |
| **Socioeconomic and geographic factors** | | | | | | | | | | |  |
| Gender | Boys | 0.65 | - |  | - | - | - |  | - | - |  |
|  | Girls | 0.42 | .64 | .15 | .48 | .86 | .82 | .15 | .61 | 1.09 | .18 |
| Maternal/female carer education level | No education | 0.67 | - |  | - | - | - |  | - | - |  |
|  | Primary | 0.72 | 1.06 | .20 | .70 | 1.58 | 1.08 | .21 | .71 | 1.63 | .72 |
|  | Secondary | 0.41 | .61 | .20 | .44 | .90 | .72 | .22 | .46 | 1.11 | .14 |
|  | Higher education | 0.33 | .49 | .25 | .23 | .80 | .71 | .30 | .39 | 1.30 | .26 |
| Paternal/male carer education level | No education | 0.62 | - |  | - | - | - |  | - | - |  |
|  | Primary | 0.76 | 1.21 | .24 | .77 | 1.95 | 1.23 | .24 | .76 | 1.97 | .40 |
|  | Secondary | 0.58 | .94 | .23 | .60 | 1.48 | 1.21 | .25 | .73 | 1.97 | .46 |
|  | Higher education | 0.28 | .45 | .24 | .28 | .73 | .75 | .29 | .42 | 1.31 | .30 |
| SES | | - | .97 | .01 | .95 | .99 | .97 | .01 | .95 | .99 | .003 |
| **Environmental** | | | | | | | | | | | |
| Home has any outside space | No | 0.75 | - |  | - | - | - |  | - | - |  |
|  | Yes | 0.45 | .60 | .16 | .44 | .83 | .86 | .17 | .63 | 1.20 | .37 |
| Number of bedrooms | |  | .80 | .07 | .70 | .91 | .89 | .06 | .78 | 1.01 | .06 |
| Number of tobacco selling shops in the neighbourhood | |  | 1.04 | .02 | 1.0 | 1.09 | 1.02 | .02 | .98 | 1.07 | .33 |
| **Smoking related behaviours** | | | | | | | | | | | |
| Smoking restrictions to smokers in the household and visitors | Complete restriction | 0.48 | - | - | - | - | - | - | - | - |  |
|  | Partial or no restriction | 0.57 | 1.19 | .15 | .89 | 1.60 | 1.45 | .20 | .98 | 21.4 | .06 |
| Smoker in the household allowed to smoke in front of children | No | 0.51 | - | - | - | - |  |  |  |  |  |
|  | Yes | 0.54 | 1.07 | .15 | .80 | 1.45 | .87 | .20 | .58 | 1.30 | .49 |
| Visitors allowed to smoke in front of children | No | 0.50 | - |  | - | - | - | - | - | - |  |
|  | Yes | 0.57 | 1.13 | .16 | .82 | 1.57 | .86 | .21 | .57 | 1.30 | .47 |
| Anyone smokes inside car | No | 0.50 | - |  | - | - |  |  |  |  |  |
|  | Yes | 0.56 | 1.13 | .15 | .84 | 1.52 | 1.11 | .14 | .84 | 1.48 | .46 |
| Near someone smoking other than home and car | No | 0.82 | - |  | - | - | - | - | - | - | - |
|  | Yes | 0.50 | .61 | .24 | .38 | .97 | .78 | .24 | .49 | 1.25 | .29 |
| ^a^Observed geometric mean cotinine.  ^b^Regression coefficients have been exponentiated to represent multiplicative effect on cotinine levels associated with unit increase in possible predictor variables. For categorical predictors, it describes a multiplicative change compared with the reference category.  *Estimates of SHS exposure for each variable while adjusting for all other variables in the model. | | | | | | | | | | | |

| **Table 3 Regression analysis of explanatory variables of SHS exposure (log cotinine) in non-smoking primary school children excluding possible tobacco users** | | | | | | | | | | | |
| --- | --- | --- | --- | --- | --- | --- | --- | --- | --- | --- | --- |
| **Variable** | | **Mean cotinine^a^** | **Regression coefficients (unadjusted)** | | | | **Regression coefficients (adjusted)*** | | | | |
|  |  |  | **Exp estimate^b^** | **SE** | **95% CI** | | **Exp estimate^b^** | **SE** | **95% CI** | | **P value** |
| Saliva cotinine levels | | 0.35 | **-** |  | **-** | |  |  |  |  |  |
| **Socioeconomic and geographic factors** | |  | | | | | | | | | |
| Gender | Boys | 0.40 | - |  | - | - | - |  | - | - |  |
|  | Girls | 0.31 | .78 | .09 | .65 | .92 | .88 | .08 | .75 | 1.03 | .11 |
| Maternal/female carer education level | No education | 0.50 | - |  | - | - | - |  | - | - |  |
|  | Primary | 0.42 | .84 | .13 | .65 | 1.10 | .95 | .13 | .73 | 1.23 | .73 |
|  | Secondary | 0.32 | .64 | .13 | .50 | .83 | .78 | .14 | .59 | 1.03 | .08 |
|  | Higher education | 0.24 | .47 | .15 | .35 | .64 | .76 | .17 | .54 | 1.06 | .10 |
| Paternal/male carer education level | No education | 0.50 | - |  | - | - | - |  | - | - |  |
|  | Primary | 0.47 | .94 | .16 | .68 | 1.30 | 1.07 | .16 | .78 | 1.48 | .67 |
|  | Secondary | 0.36 | .72 | .16 | .53 | .98 | .98 | .16 | .70 | 1.35 | .90 |
|  | Higher education | 0.24 | .48 | .16 | .35 | .68 | .78 | .18 | .54 | 1.10 | .16 |
| SES | | - | .98 | .01 | .97 | .99 | .99 | .01 | .98 | .99 | .01 |
| **Environmental** | |  | | | | | | | | | |
| Home has any outside space | No | 0.41 | - |  | - | - | - |  | - | - |  |
|  | Yes | 0.33 | .79 | .09 | .66 | .95 | .91 | .09 | .76 | 1.08 | .31 |
| Number of bedrooms | |  | .87 | .04 | .82 | .95 | .96 | .04 | .89 | 1.03 | .26 |
| Number of tobacco selling shops in the neighbourhood | |  | 1.05 | .01 | 1.03 | 1.08 | 1.05 | .01 | 1.02 | 1.07 | .0002 |
| **Smoking related behaviours** | |  | | | | | | | | | |
| Lives with smoker | No | 0.27 | - |  | - | - | - |  | - | - |  |
|  | Yes | 0.50 | 1.84 | .08 | 1.57 | 2.18 | 2.01 | .12 | 1.60 | 2.53 | <.0001 |
| Smoking restrictions to smokers in the household and visitors | Complete restriction | 0.45 | - |  | - | - | - | - | - | - |  |
|  | Partial or no restriction | 0.33 | .73 | .11 | .59 | 1.11 | 1.26 | .14 | .95 | 1.65 | .10 |
| Visitors allowed to smoke in front of children | No | 0.33 | - |  | - | - | - | - | - | - |  |
|  | Yes | 0.46 | 1.38 | .12 | 1.09 | 1.72 | .88 | .13 | .68 | 1.14 | .32 |
| Anyone smokes inside car | No | 0.36 | - |  | - | - |  |  |  |  |  |
|  | Yes | 0.33 | .96 | .10 | .80 | 1.14 | 1.0 | .08 | .85 | 1.17 | 1.0 |
| Near someone smoking other than home and car | No | 0.40 | - |  | - | - | - | - | - | - |  |
|  | Yes | 0.34 | .86 | .13 | .66 | 1.11 | .86 | .12 | .67 | 1.10 | .23 |
| ^a^Observed geometric mean cotinine  ^b^Regression coefficients have been exponentiated to represent multiplicative effect on cotinine levels associated with unit increase in possible predictor variables. For categorical predictors, it describes a multiplicative change compared with the reference category.  *Estimates of SHS exposure for each variable while adjusting for all other variables in the model. | | | | | | | | | | | |
